# The European Pine Marten *Martes martes* (Linnaeus, 1758) Is Autochthonous in Sicily and Constitutes a Well-Characterised Major Phylogroup within the Species (Carnivora, Mustelidae)

**DOI:** 10.3390/ani12192546

**Published:** 2022-09-23

**Authors:** Luca Vecchioni, Federico Marrone, Simone Costa, Calogero Muscarella, Elena Carra, Vincenzo Arizza, Marco Arculeo, Francesco Paolo Faraone

**Affiliations:** 1 Department of Biological, Chemical and Pharmaceutical Sciences and Technologies (STEBICEF), University of Palermo, Via Archirafi 18, 90123 Palermo, Italy; 2 Cooperativa Silene, Via D’Ondes Reggio 8a, 90127 Palermo, Italy; 3 Independent Researcher, Viale Regione Siciliana S. E. 532, 90129 Palermo, Italy

**Keywords:** Pleistocene refugia, biodiversity on islands, mitochondrial DNA, Quaternary glaciations

## Abstract

**Simple Summary:**

The faunal assemblages currently occurring on islands are often a mélange of native and non-native species, and the actual status of some of them is doubtful at present. Since different laws are enforced for native and non-native species, sound knowledge about their status is pivotal for both their management and our understanding of the natural history of the studied areas. In the frame of this work, the Sicilian population of the European pine marten is genetically characterized for the first time, based on mitochondrial DNA sequences. Our results prove that the European pine marten is native on the island, where it arrived during the Pleistocene, and is represented there by a well-differentiated and endemic evolutionary lineage.

**Abstract:**

No molecular data are currently available for the Sicilian populations of the European pine marten *Martes martes*, thus preventing any sound inference about its native or non-native status on the island, as well as the local phylogeography of the species. In order to investigate these issues, we sequenced two mtDNA markers in road-killed specimens collected in Sicily. Both markers consistently demonstrated the existence of a well-characterised Sicilian clade of the species, which is endemic to the island and constitutes the sister group of a clade including the Mediterranean and Central–North European major phylogroups of the European pine marten. Such evidence supports the autochthony of *Martes martes* in Sicily and points to a natural Pleistocene colonisation of the island followed by isolation. The occurrence of a, to date undetected, major phylogroup of the species in Sicily calls for the dedicated monitoring of the Sicilian populations of the species in order to preserve this evolutionarily significant unit.

## 1. Introduction

The recent mustelid fauna of Sicily includes one extinct and two extant species: otter *Lutra lutra* (Linnaeus, 1758), which disappeared in Sicily at the beginning of the XX century [1], weasel *Mustela nivalis* Linnaeus, 1766, and European pine marten *Martes martes* (Linnaeus, 1758), with the last two both widespread throughout the island in natural and anthropogenic habitats [2,3]. Conversely, badger *Meles meles* (Linnaeus, 1758) and beech marten *Martes foina* (Erxleben, 1777), although occurring in neighbouring southern Calabria, have never been reported to occur in Sicily [4].

European pine marten, *Martes martes* (Linnaeus, 1758), is currently widespread from the Iberian Peninsula to Western Siberia, including Great Britain, Ireland, Anatolia, the Caucasus, and parts of Iran, Iraq, and Syria [5,6,7]. The species also occurs on some Mediterranean islands such as Sardinia, Corsica, Elba, Sicily, and the Balearic Islands (Majorca and Minorca) [8,9]. The conservation status of the European pine marten has been evaluated as “Least Concern” both on a global scale [6] and in Italy [10].

The Mediterranean populations of *M. martes* have been attributed, based on morphology, to three endemic subspecies, i.e., *M. martes latinorum* (Barret-Hamilton 1904) from Sardinia and the Balearic Islands, *M. m. minoricensis*, Alcover, Delibes, Gosálbez and Nadal 1987, from Minorca, and *M. m. notialis* (Cavazza 1912) from Sicily and southern Italy. The validity and distribution of each subspecies have been debated over time [8] as well as the native status of *M. martes* in these islands. Based on historical documents, the species is currently considered as introduced by man on some of the islands [6,11,12]. The subspecies occurring in Sicily and southern Italy is currently considered synonymous with the nominotypical subspecies [13], but this has never been verified by genetic evidence.

Molecular studies on the phylogeny of *Martes martes* [14,15,16] have highlighted the existence of three major phylogroups within the species: the Mediterranean, Central–Northern European and Fennoscandian–Russian ones. This phylogeographic pattern likely emerged during the Quaternary, with the recolonization of Northern Europe from Mediterranean and non-Mediterranean refugia and possibly also from cryptic refugia located in the Carpathians [15]. In this context, among Mediterranean islands, only samples from Sardinia and Elba Island were to date analysed using molecular tools, and they proved to belong to the Mediterranean phylogroup, showing haplotypes shared with, or scarcely diversified from, mainland European pine marten samples [15], but see also [17]. 

Lacking genetic data about Sicilian *Martes martes* populations, we sequenced two mitochondrial DNA markers in European pine marten specimens sampled on the island with the explicit aim of investigating the native or non-native status of the species in Sicily, and its relationships with other mainland and island populations.

## 2. Materials and Methods

Muscle tissue samples were collected from four road-killed *Martes martes* specimens found in Sicily and identified in situ based on Genovesi & De Marinis [18]. Total DNA was extracted using the BIORON GmbH “Ron’s Tissue DNA Mini Kit”, following the standard protocol provided by the producer. Fragments of two mitochondrial DNA markers (mtDNA) were amplified: (i) the region which includes part of the cytochrome *b* gene, *tRNAPro*, *tRNAThr*, the control region, and the initial part of *12S* rRNA (hereafter referred to as “mtDNA I”), using the primer pair “LutbF” and “LLU12SH91” [19]; and (ii) the cytochrome c oxidase subunit I, *COI* (hereafter “mtDNA II”), using the universal primer pair “LCO1490” and “HCO2198” [20].

The PCR mix and thermal cycles for amplifying the “mtDNA I” fragment were conducted following the protocol described by Pertoldi et al. [19]. The PCR mix for amplifying the “mtDNA II” fragment consisted of 18.3 μL double-distilled water, 2.5 μL Buffer 10X including 15 mM MgCl_2_ solution, 0.6 μL dNTPs (10 mM of each), 0.6 μL of each primer (10 μM), 0.4 μL BIORON DFS-Taq DNA Polymerase 5 U/μL, and 2 μL of DNA template, for a total volume of 25 μL. The thermal cycle consisted of a 4 min denaturation step at 94 °C, followed by 35 cycles of denaturing (94 °C for 40 s), annealing (48 °C for 40 s), and extension (72 °C for 1 min), followed by a final extension step at 72 °C for 7 min.

After PCRs, five μL of each PCR product was used to perform electrophoresis on 2% agarose gel. The outcome of the electrophoresis was verified using a UV transilluminator. The samples that showed a single clear band with the expected length for the marker used were purified using the Exo-SAP-IT^®^ kit (Affymetrix USB). Sequencing was conducted by Macrogen Inc. (Madrid, Spain) with an ABI 3130xL sequencer. The primers used for the PCRs were also used for the sequencing of the PCR products. The quality of the obtained chromatograms was verified through their “Phred scores” [21], keeping only those sequences that showed high-quality bases (QV > 20). The novel *Martes martes* sequences were deposited in the public database GenBank (see Table 1 for their accession numbers, A.N.).

**Figure 1:**
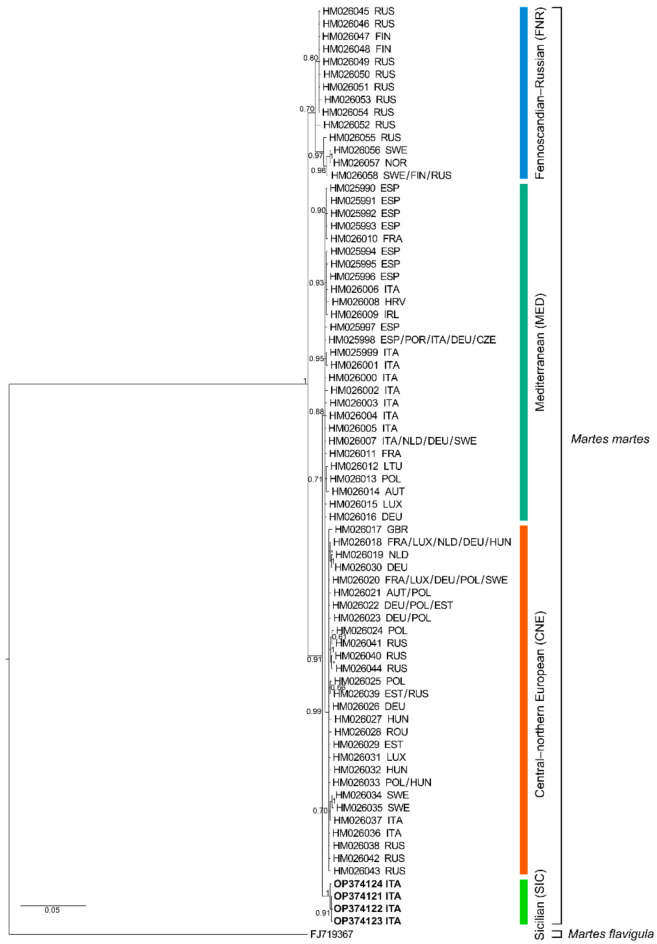
Bayesian phylogram (50% majority rule consensus tree) for *Martes* spp. based on the 1563-bp long “mtDNA I” fragment. A sample of *Martes flavigula* was used as outgroup. Node statistical support is reported as nodal posterior probabilities. Asterisks indicate posterior probability values lower than 50. Rectangles refer to the major phylogroups identified by Ruiz-González et al. [15] [i.e., Mediterranean (MED), Central–Northern European (CNE), Fennoscandian–Russian (FNR) phylogroups], plus the novel Sicilian phylogroup (SIC). Square brackets group the samples according to the current taxonomy of the genus. Our novel sequences are reported in bold. The three-letter country codes shown in the figure are reported according to the “ISO 3166-1 alpha-3 standard.

**Figure 2:**
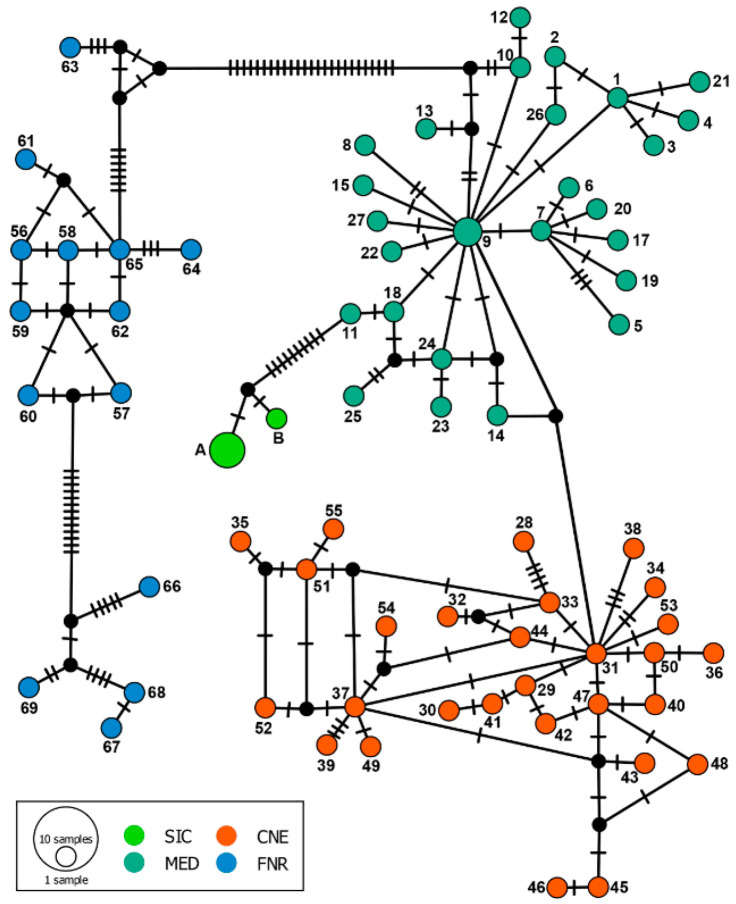
Median-joining haplotype network based on the “mtDNA I” dataset of *Martes martes*. Dashes indicate substitutions steps. Each circle represents a haplotype, and its size is proportional to its frequency. Numbers close to haplotypes refers to those reported by Ruiz-González et al. [15]. Novel Sicilian haplotypes are coded as “A” (A.N.: OP374121—OP374123) and “B” (A.N.: OP374124). Colours refer to phylogroups shown in Figure 1. (Sicily (SIC), Mediterranean (MED), Central–Northern European (CNE), Fennoscandian–Russian (FNR) phylogroups).

**Figure 3:**
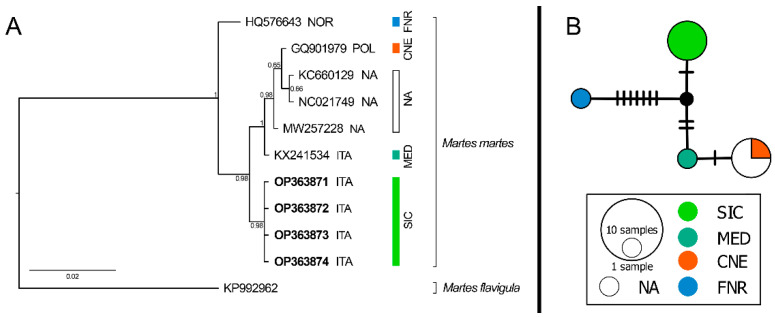
(**A**) Bayesian phylogram (50% majority rule consensus tree) for *Martes* spp. based on a 646-bp “mtDNA II” fragment. A sample of *Martes flavigula* was used as outgroup. Node statistical support is reported as nodal posterior probabilities. Rectangles refer to phylogroups identified by Ruiz-González et al. [15] (see Figure 1 for their codes), plus the novel Sicilian phylogroup (SIC). NA, Geographical origin of the samples not available. Square brackets group the samples according to the current taxonomy of the genus. Our novel sequences are reported in bold. The three-letter country codes shown in the figure are reported according to the “ISO 3166-1 alpha-3 standard”. (**B**) Median-joining haplotype network based on the “mtDNA II” dataset of *Martes martes*. Dashes indicate substitutions steps. Each circle represents a haplotype, and its size is proportional to its frequency. Colours refer to phylogroups shown in (**A**).

In order to compare the new sequences (four for each mtDNA dataset) with those publicly available, 75 *Martes martes* mtDNA sequences (69 for the “mtDNA I” and 6 for the “mtDNA II” datasets) and 2 of *M. flavigula* (Boddaert, 1785) (to be used as outgroups) were downloaded from GenBank and included in the analyses (see Figure 1, Figure 2 and Figure 3B for their A.N.). All sequences were aligned, independently for each mtDNA dataset, with the software MEGAX [22] using the ClustalW method [23], and the relationships among haplotypes were assessed based on Bayesian inference (BI) using the software package MrBayes v. 3.2.6 [24]. The best evolutionary model was selected among those analysed by MrBayes using Bayesian model choice criteria (nst = mixed, rates = gamma). Two independent Markov Chain Monte Carlo analyses were carried out with 1 million generations each (temperature: 0.2; default priors). Trees and parameter values were sampled every 100 generations, resulting in 10,000 trees for each analysis. Convergence of chains was assessed to ensure proper mixing (effective sample Size > 200 in all the analyses). The initial 25% of trees were discarded as “burn-in”. Posterior probability values were reported as nodes support.

The haplotype networks including all the analysed *Martes martes* sequences were built for both mtDNA datasets, using the software PopART v. 1.7 by the University of Otago, New Zealand (http://popart.otago.ac.nz (accessed on 20 August 2022)), implementing the median-joining network algorithm as suggested by Bandelt et al. [25].

## 3. Results

**Figure A1:**
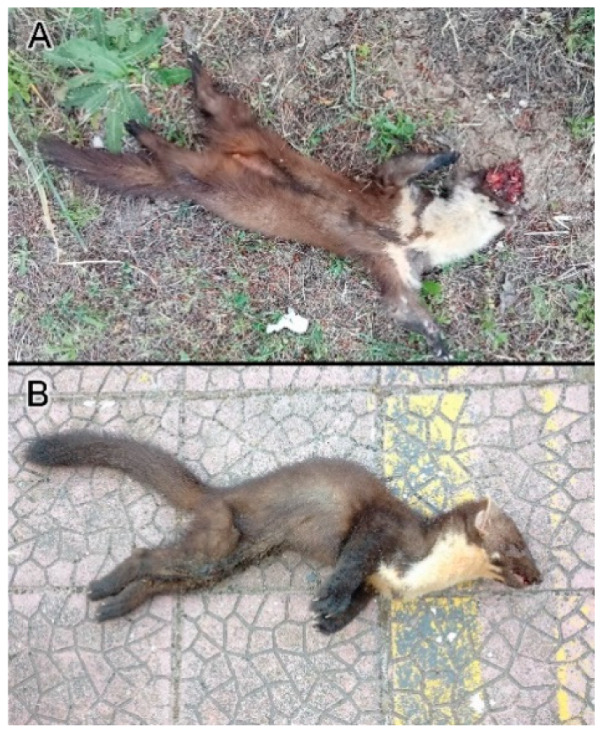
Road-killed European pine marten specimens collected in the frame of this work. (**A**) Specimen of *Martes martes* from “C.da Strasatto”; (**B**) Specimen of *M. martes* from “C.da Misericordia” (see Table 1 for further information).

Overall, four road-killed *Martes martes* specimens were collected (Table 1 and Figure A1). Novel mtDNA sequences belonging to Sicilian *M. martes* were produced for both selected markers. The inferred phylogenetic trees, based on a 1563 and 646 base-pair long fragments (mtDNA I” and “mtDNA II”, respectively) included both new and published sequences and were rooted on the congeneric *M. flavigula* (A.N. FJ719367 and KP992962, respectively). Both trees showed a well-supported topology clustering in the sequences of *M. martes* in monophyletic clades, thus confirming the morphology-based identification of the Sicilian specimens (Figure 1 and Figure 3B).

With regard to the “mtDNA I” dataset and in accordance with Ruiz-González et al. [15], the occurrence of the Mediterranean, Central–Northern European and Fennoscandian–Russian phylogroups was observed; in addition, the Sicilian *Martes martes* sequences clustered together in a well-supported clade and showed a sister relationship to a clade including the Mediterranean and Central–North European major phylogroups (Figure 1).

In accordance with the phylogenetic analysis, the “mtDNA I” haplotype network showed a clear separation among the four different major phylogroups (Figure 2). The genetic uncorrected “*p*” distances between the Sicilian clade and the previously identified phylogroups ranged from 1.05 to 2.65% and were comparable to the distances among all the major phylogroups included in the analysis (i.e., 0.63–2.49%).

Similarly to what has been observed in the “mtDNA I” dataset, and despite the limited number of available sequences to be used for comparison, the “mtDNA II” dataset showed a clear separation of the four phylogroups, grouping all the Sicilian sequences together in a novel major phylogroup (Figure 3).

## 4. Discussion

The current Sicilian Mammalian fauna originated following three main colonisation routes: (1) colonisation from northern Africa during late Miocene to the early Pliocene [26]; (2) colonisation from the Italian peninsula during the Pleistocene, from the early stages of this epoch [27,28,29,30] to the transition between its later stages and the early Holocene [31,32,33]; and (3) ancient [34,35,36,37] and recent [2] introductions by man. The second pattern is linked to the glacial and palaeogeographical events that occurred during the Pleistocene, which are known to have played a crucial role in shaping the current composition of the Italian biota [38,39].

Based on available data, the anthropogenic introduction of *Martes martes* in Sicily can be ruled out, while Pleistocene glacial fluctuations and marine regressions seem to have been decisive for the origin of the Sicilian population of the species. In fact, we detected a clear differentiation of the Sicilian samples, which form a monophyletic unit endemic to Sicily, which is not nested in any of the main phylogroups detected by Ruiz-González et al. [15] and, indeed, constitutes a to-date undetected fourth major phylogroup of the species (Figure 1). This is also corroborated by the uncorrected “*p*” distances, based on the “mtDNA I” dataset, between the Sicilian clade and the Mediterranean (1.04%) and Central–Northern European phylogroups (1.21%), which do not have dissimilar values to each other. The Pleistocene origin of the Sicilian population of *M. martes* is supported by the fossil record of the island, which indicates that the oldest Sicilian fossils likely attributable to this species can be dated to the late Pleistocene [40,41], and remains are also present from the ancient and recent Holocene [41].

The colonization of Sicily through Pleistocene land bridges also occurred for other carnivores such as the wild cat, *Felis silvestris* Schreber, 1777 [31], and the recently extinct wolf, *Canis lupus* Linnaeus, 1758 [32]. Conversely, current Sicilian populations of the weasel, *Mustela nivalis*, and of the red fox, *Vulpes vulpes* Linnaeus, 1758, seem to have a more complex origin since, despite both occur in the Pleistocene fossil record of Sicily [41], their genetic characteristics suggest a probable ancient man-mediated introduction [36,37].

The translocation of small carnivores for fur exploitation and/or rodent control in the Mediterranean islands is, moreover, well documented by various historical documents, which report introductions even in islands where these species are currently absent, as in the Tuscan Archipelago [11]; however, molecular data exclude this origin for European pine marten in Sicily.

## 5. Conclusions

Our results prove the existence of a well-characterised major phylogroup of *Martes martes* in Sicily, which is, to date, to be considered endemic to the island. Such a pattern is already known for other native Sicilian vertebrates [27,28,29,30,31,32,33,42,43], and seems linked to the long-term Quaternary absence of gene flow between Sicilian and southern peninsular Italian populations of these species, which led to a sharp differentiation of the insular populations. However, it should be stressed that, based on available data, we cannot currently exclude that the Sicilian *M. martes* populations might be identical or only scarcely differentiated from those occurring in southern Calabria, as it has been observed in other vertebrate species such as the Apennine hare, *Lepus corsicanus* de Winton, 1898 [33], and several amphibians and reptiles (e.g., [44,45,46,47,48]). Accordingly, in order to better understand the phylogeography of the European pine marten and draw a well-defined picture of the timing of its expansion towards the island (or vice versa), it is desirable to further expand current analyses by including samples from southern peninsular Italy, for which no data are available (see Ruiz-González et al. [15]).

The occurrence of an endemic major phylogroup of the European pine marten in Sicily calls for the dedicated monitoring of the Sicilian populations of the species, in order to preserve this evolutionary significant unit, and their fine-scale molecular characterisation to point out the possible occurrence of independent management units within the island. Moreover, obtaining information about the nuclear genome of the species throughout its distribution range is desirable in order to confirm the observed mitochondrial DNA diversity pattern and the phylogeography of the species.

## Figures and Tables

**Table 1:** Origin and GenBank accession numbers (A.N.) of the analysed Sicilian *Martes martes* specimens. Geographical coordinates are expressed in terms of decimal degrees (Map Datum: WGS84).

Sampling Date	Location	Province	Coordinates	Elevation (m a.s.l.)	“mtDNA I” A.N.	“mtDNA II” A.N.
25 August 2016	SS186, Monreale	Palermo	38.076972° N, 13.290619° E	245	OP374121	OP363871
22 July 2021	SP157, C.da Calagni	Messina	38.030690° N, 14.821550° E	494	OP374122	OP363872
2 August 2021	SS121, C.da Misericordia	Enna	37.580474° N, 14.283588° E	663	OP374123	OP363873
22 May 2022	SS624, C.da Strasatto	Palermo	38.008194° N, 13.242387° E	779	OP374124	OP3638714

## Data Availability

Produced sequences are available on GenBank (https://www.ncbi.nlm.nih.gov/genbank/).
